# Validity of a web-based dietary questionnaire designed especially to measure the intake of phyto-oestrogens

**DOI:** 10.1017/jns.2016.28

**Published:** 2016-09-09

**Authors:** Sanna Nybacka, Heléne Bertéus Forslund, Maria Hedelin

**Affiliations:** 1 Department of Internal Medicine and Clinical Nutrition, Institute of Medicine, Sahlgrenska Academy, University of Gothenburg, Gothenburg, Sweden; 2 Department of Oncology, Institute of Clinical Sciences, Division of Clinical Cancer Epidemiology, Sahlgrenska Academy, University of Gothenburg, Gothenburg, Sweden

**Keywords:** Diet assessment, Alkylresorcinol metabolites, Phyto-oestrogens, Validation, Web-based research, DQ, diet questionnaire, ER-β, oestrogen receptor-β, FR, food record

## Abstract

A diet questionnaire (DQ) designed to assess habitual diet and phyto-oestrogen intake was developed. This study aimed to examine the validity of the DQ in men, with and without having prostate cancer. The DQ was validated against alkylresorcinol metabolites measured in urine as objective biomarkers of whole grain wheat and rye (WG) intake, and a 4-d estimated food record (FR) was used for relative comparison. Participants (*n* 61) completed both methods and provided spot urine samples. We found a statistically significant correlation between the DQ and FR for reported whole grain intake and isoflavonoids, as well as for intake of macronutrients, except protein. The correlation coefficient between the two methods was on average *r* 0·30, lowest for lignans (*r* −0·11) and highest for alcohol (*r* 0·65). Reported energy intake was lower in the DQ compared with FR (8523 *v.* 9249 kJ (2037 *v*. 2211 kcal), respectively; *P* = 0·014). Bland–Altman plots showed an acceptable agreement; most cases were within the limits (95 % CI) of agreement on reported energy intake, as well as intake of macronutrients, except protein (which was underestimated in the DQ compared with the FR). The correlation of alkylresorcinol with WG intake was statistically significant in the DQ (*r* 0·31, *P* = 0·015), but not in the FR (*r* 0·18, *P* = 0·12) and the weighted κ was 0·29 and 0·11, respectively. In conclusion, the results showed that the DQ have a reasonable validity for measuring WG intake and most nutrients, and, after some adjustments regarding protein intake assessment have been made, the DQ will be a promising tool.

In nutrition research, various dietary components are explored in order to study relationships between dietary exposures and disease outcome. However, it is well known that the various subjective dietary assessment methodologies used in nutrition research have limitations, which we need to cater for. To be able to investigate the diet–disease association, the dietary assessment tool of choice needs to capture the dietary components of interest in a satisfactory manner.

In Europe, 417 000 new cases of prostate cancer were identified in 2012, and it is the most common cancer among men in Westernised countries^(^^1^^)^. Many appear to be willing to alter their diet in hope of decreasing the tumour growth rate, and we have previously shown that 50 % of all men with prostate cancer choose to self-medicate with a variety of dietary supplements upon no scientific basis^(^^2^^)^. In the absence of data with high-grade evidence on how the diet is affecting prostate tumour growth, no adequate dietary advice can be given to these patients.

A diet rich in phyto-oestrogens (e.g. soya beans, rye bran and flaxseeds) has been shown to reduce the risk of developing prostate cancer^(^^3^^–^^8^^)^, especially among individuals with a particular genetic type of the oestrogen receptor-β gene (ER-β)^(^^9^^)^. ER-β has demonstrated a cancer-inhibiting effect and is expressed in normal prostate epithelium, but the level of expression decreases gradually during the development of prostate cancer. Since phyto-oestrogens are structurally similar to female sex hormones and bind to ER-β with high affinity, phyto-oestrogens and ER-β should be able to interact during the development of cancer^(^^10^^,^^11^^)^. This association needs to be further investigated in order to draw any conclusions on the effect of phyto-oestrogen intake and the progress of prostate cancer.

To meet the demand of convenient and updated tools for assessing dietary intake in large-scale studies, we developed a new web-based diet questionnaire (DQ) based on two previously validated paper-based questionnaires^(^^12^^,^^13^^)^. The questionnaire was designed to measure the habitual diet, and specifically the intake of phyto-oestrogen-rich foods, while at the same time covering the food items on the market today. The questionnaire is planned to be used in an intervention study to measure dietary intake in a group of men with prostate cancer, but also in the general population. Therefore, this study aimed to examine the validity of the questionnaire against a reference method (a 4-d diet record), in elderly men with and without prostate cancer. Also, alkylresorcinol metabolites measured in urine were used as objective markers of whole grain wheat and rye intake. To ensure that the questions in the DQ were correctly understood, a ‘face-to-face’ validation was conducted with the first ten study participants.

## Materials and methods

### Study population

Two groups of men were recruited during the period of March 2011 to June 2011; one including men with prostate cancer from the Department of Urology, Sahlgrenska University Hospital, Gothenburg, Sweden and one including randomly selected men from the Swedish population register. The inclusion criteria were men aged 57–71 years from the healthcare region of Västra Götaland, Sweden. The exclusion criteria were: treatment with antibiotics during the last 3 months (due to the metabolism of phyto-oestrogens), other severe mental or physical illness, food allergy or inability to understand written Swedish. Men from the population register were excluded if they had ever been diagnosed with cancer.

All men with prostate cancer who met the inclusion criteria were informed and received written information about the study by the treating physician; fifty-one persons in total. In total, thirty-eight men agreed to participate.

**Fig. 1.**
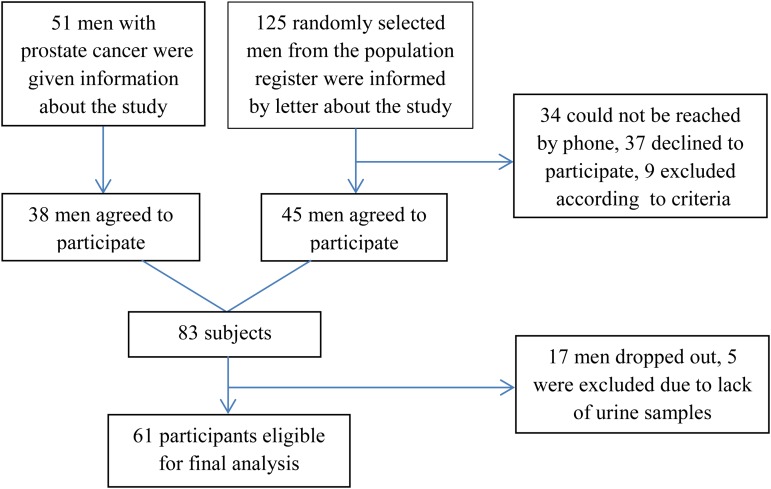
Flowchart of study participant selection procedure.

A total of 125 randomly selected men from the population register were informed about the study by letter and later by telephone; forty-five men agreed to participate in the study. Of the initial eighty-three participants (both groups), sixty-one participants were eligible in the final analyses (Fig. 1).

### Study protocol

All participants enrolled were provided with a urine sampling kit, written information about the study and a booklet to keep a food record (FR) in. Participants were scheduled for a visit at the study centre at the Department of Internal Medicine and Clinical Nutrition, Sahlgrenska Academy, Sweden and instructed on how to keep a FR and to collect a spot urine sample the morning after completing the FR. At the study centre all participants signed a written consent form and filled in the DQ. All FR were reviewed by the study dietitian during the visit to ensure that they were properly filled in, as was the DQ. The first ten study participants underwent a face-to-face interview to ensure that the DQ was perceived correctly. This study was conducted according to the guidelines laid down in the Declaration of Helsinki and all procedures involving human subjects were approved by the Gothenburg Regional Ethics Committee (no. 765–10).

### Dietary assessments

#### Diet questionnaire

Based on a previously developed and validated paper-based DQ that has been used in several studies, for example, the Swedish Obese Study and the Cancer of the Prostate in Sweden (CAPS) study^(^^12^^,^^13^^)^, we further developed the questions regarding food intake. The food items and dishes that are now included are more in line with current food trends and have a better coverage of phyto-oestrogen-rich foods.

The new web-based DQ was designed to capture eating habits during the past 3 months, as well as questions regarding lifestyle, such as physical activity, intake of food supplements, alcohol consumption, tobacco use and use of medication (i.e. antibiotics and diabetes medications). The questionnaire also included questions on socio-economic parameters and anthropometric measurements. An extended version of the questionnaire was developed for men with prostate cancer with questions regarding family history of prostate cancer, and if their lifestyle habits or intake of dietary supplements had changed after being diagnosed with cancer.

**Fig. 2.**
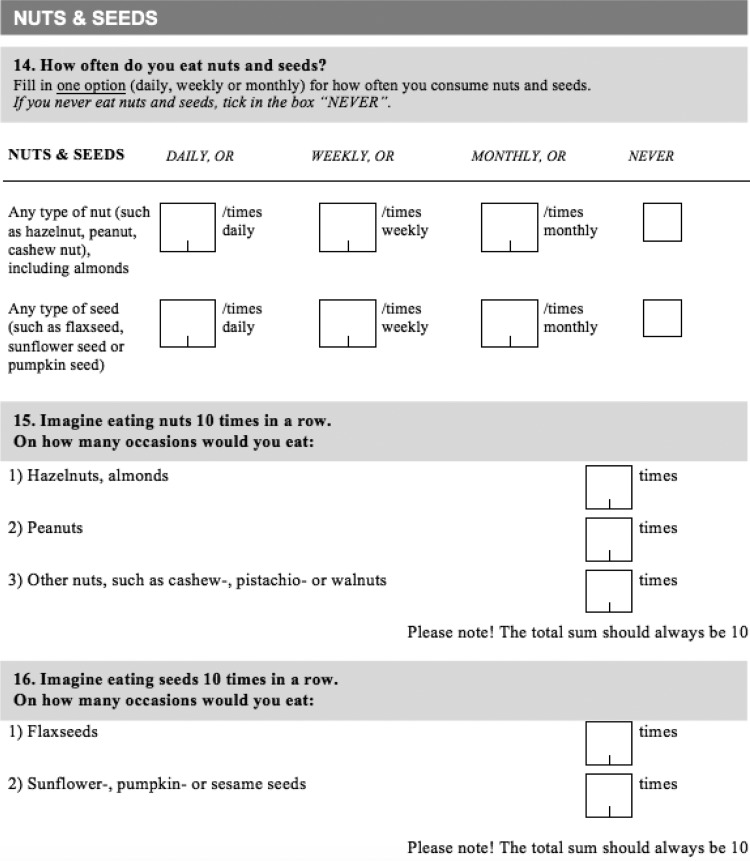
Diet questionnaire: nuts and seeds section.

In total, 184 food items and complex dishes were included. The questions regarding dietary intake were divided into twelve food group categories as follows: sandwiches; porridge and muesli; nuts and seeds; drinks; vegetables and fruit; meat and meat products; fish and seafood; vegetarian dishes and eggs; potatoes, pasta and grains; and candy, cakes and snacks. Within each food group, the frequency of intake was listed first with optional answering frequencies divided into: daily; weekly; monthly or never. As a follow-up question, food items were specified within each food group category and ranked according to intake during the last ten eating occasions. For an example, see the nuts and seeds section of the questionnaire (Fig. 2). Meal sizes were calculated using standard portion sizes, based on information from the Swedish National Food Agency.

#### 4-d Food record

An estimated 4-d FR was completed as a reference method. Participants were asked to keep record of all foods and drinks consumed during four consecutive days, including a weekend day. Meal size was estimated by describing the amounts in grams or household measurements, such as tablespoons, decilitres, glasses, bowls or pieces. Participants were also asked to fill in as detailed information as possible regarding the manufacturers’ name (on pre-cooked dishes), cooking methods and fats used for cooking.

#### Energy and nutrient calculation

Intake of energy and specific nutrients, as well as phyto-oestrogens, was estimated by linking information from the DQ and 4-d FR to the Swedish National Food Agency's nutritional database (version 2010-08)^(^^13^^)^ and to a previously developed phyto-oestrogen database^(^^14^^)^. Phyto-oestrogen exposure was categorised into: total lignan intake, which included intake (μg/d) of secoisolariciresinol, matairesinol, lariciresinol, isolariciresinol, pinoresinol, syringaresinol and medioresinol; total dietary isoflavonoid intake (μg/d), which included genistein, daidzein, formononetin, biochanin A and equol. For the DQ, the calculations were carried out with a specially developed calculation software. For the 4-d FR, the software Dietist XP version 3.1 was used^(^^15^^)^. The reported intakes of whole grain wheat and rye were calculated using a table presenting the whole grain content of various foods, provided by the Swedish National Food Agency. All individual nutrient values were calculated as average intake per d.

### Biomarker

#### Urinary alkylresorcinol metabolites

All men collected spot urine samples (3 × 10 ml) after an overnight fast (22.00 hours) the morning after finishing the 4-d FR. Urine samples were stored in a household freezer until the visit at the study centre, and there it was stored at −80°C until analysis. The amounts of alkylresorcinol metabolites 3,5-dihydroxybenzoic acid (DHBA) and 3-(3,5-dihydroxyphenyl)-1-propanoic acid (DHPPA) in urine were analysed by a GC-MS method according to Marklund *et al*.^(^^16^^)^ at the Swedish University of Agricultural Sciences (SLU), Department of Food Science, Uppsala. Creatinine was determined by a photometric method at the Sahlgrenska University Hospital, Laboratory Medicine, Clinical Chemistry.

### Statistical analysis

Baseline characteristics for men with and without prostate cancer were compared using two-sided *t* tests for equal means for continuous, normally distributed variables, independent-samples Mann–Whitney *U* tests for non-normally distributed variables and χ^2^ tests for categorical variables. The mean values and standard deviations, and median (25 and 75 percentile) of total energy and nutrient intake are presented. To compare unadjusted energy and nutrient intake estimated by the two methods, the 4-d FR and the DQ, the Wilcoxon signed-rank test was performed and Spearman correlation coefficients were calculated. Nutrient density was obtained by dividing the estimated nutrient intake (μg/d, mg/d, g/d) by total energy intake (MJ/d)^(^^17^^)^. Correlation analyses on urinary alkylresorcinol metabolites were adjusted for creatinine (alkylresorcinols μmol/l)/(creatinine mmol/l). Because most nutrients were not normally distributed, all variables were log transformed prior to further analyses. Pearson correlation coefficients were then used on energy-adjusted variables. Subgroup analyses were also conducted to evaluate the influence of other covariates, such as BMI, smoking status, intake of saturated fat, alcohol intake and age (cut-off set by median). The ability to rank individuals by reported intake of whole grain and phyto-oestrogens (using energy-adjusted values) was examined by dividing the study population into tertiles of dietary intakes, as for levels of alkylresorcinol metabolites. Through a cross-tabulation the Cohen's weighted κ was obtained. The absolute agreement between the two methods was evaluated with Bland–Altman plots^(^^18^^)^. The plot obtained illustrates the differences between the two nutrient intake measurements against the mean of both methods. A 95 % CI calculated as the mean difference ±1·96 sd enables the evaluation between the methods within the limits of agreement. All statistical analyses were two-sided with a significance level at *α* < 0·05. To achieve 80 % power to reject the null hypothesis if the true correlation coefficient between alkylresorcinol and whole grain intake is 0·5, the number of participants was calculated to be at least twenty-nine persons in each group (Pearson correlation, two-sided test, *α* = 5 %, *z*-approximation). Statistical analyses were performed using SPSS Statistics for Windows, version 20.0 (released 2011; IBM Corp.) and SAS 9.2 for Windows (SAS Institute, Inc.)

## Results

Characteristics of the study participants are presented in Table 1. Mean age was 65·7 years and the proportion of obese individuals (BMI >30 kg/m^2^) was 11·5 %. The proportion of current smokers was 10 % and the proportion of men with a higher degree of education corresponded to the general population in Sweden^(^^19^^)^, which was around 33 %. There were no statistically significant differences between the two groups of men in any of the background characteristic variables; hence all further analyses were performed with both groups combined.

**Table 1.** Characteristics of study population of men with and without prostate cancer (Mean values, standard deviations and ranges for continuous data; numbers and percentages for categorical data)

	All men (*n* 61)			Men with prostate cancer (*n* 30)			Men without prostate cancer (*n* 31)			
	Mean	sd	Range	Mean	sd	Range	Mean	sd	Range	*P*
Age (years)	65·7	3·7	57–71	66·1	2·9	59–70	65·1	4·4	57–71	0·51*
Weight (kg)	85·4	14·6	65–140	82·2	13·3	65–126	88·0	15·5	65–140	0·08*
Alkylresorcinols, total (μmol/l)	47·7	50·3	3·6–348·3	49·2	64·2	4·5–351·9	46·3	32·8	3·6–157·1	0·83†
DHBA (μmol/l)	10·1	9·8	0·9–61·5	10·2	11·9	1·1–61·5	10·0	7·53	0·90–35·5	0·94†
DHPPA (μmol/l)	37·7	41·1	2·7–290·4	39·0	52·6	3·4–290·4	36·4	25·4	2·7–121·6	0·81†
	*n*	%		*n*	%		*n*	%		
BMI (category)										0·09‡
Normal weight (18·5–24·9 kg/m^2^)	28	45·9		18	60·0		10	32·3		
Overweight (25–29·9 kg/m^2^)	26	42·6		10	33·3		16	51·6		
Obese (>30 kg/m^2^)	7	11·5		2	6·7		5	16·1		
Smoking										0·98‡
Current smoker	6	9·8		3	10·0		3	9·7		
Previous smoker	30	49·2		15	50·0		15	48·4		
Non-smoker	25	41·0		12	40·0		13	41·9		
Education§										0·93‡
Compulsory school	18	30·0		8	26·7		10	32·3		
Upper secondary school	22	36·7		11	36·7		11	35·5		
University or college degree	20	33·3		10	33·3		10	32·3		

DHBA, 3,5-dihydroxybenzoic acid; DHPPA, 3-(3,5-dihydroxyphenyl)-1-propanoic acid.

* Independent-samples *t* test.

† Independent-samples Mann–Whitney *U* test.

‡ *χ*^2^.

§ Missing data *n* 1.

Mean average daily intakes of dietary variables estimated by the two different methods (DQ and FR) are shown in Table 2. Reported energy intake was lower measured by the DQ compared with FR (8523 *v*. 9249 kJ/d (2037 *v*. 2211 kcal/d); *P* = 0·014, respectively). Of the macronutrients, the largest discrepancy was shown for protein where the reported mean daily intake was on average 20 g lower in the DQ compared with the FR. Reported intake of whole grains (wheat and rye) was higher in the DQ compared with the FR (48 *v*. 34 g; *P* < 0·001). Reported daily intake of total phyto-oestrogens was higher in the FR compared with the DQ (*P* < 0·001). Rye bread contributed most to the phyto-oestrogen intake for both methods (91 % of the intake in FR and 85 % in DQ) and the estimated median intake of rye bread was 70 g/d in FR and 41 g/d in DQ. Reported intake of phyto-oestrogens did not differ between men with or without prostate cancer estimated by either of the methods (data not shown) and nearly all phyto-oestrogens (99 %) were derived from lignans.

**Table 2.** Average daily intake of energy, macronutrients, alcohol, whole grains† and micronutrients for the 4-d food record (FR) and diet questionnaire (DQ), and difference between the methods (Mean values, standard deviations, medians, and 25 and 75 percentiles for intakes; percentages, *P*, and crude and energy-adjusted (EA) correlations for difference between the methods)

	4-d FR				DQ				Difference between the methods			
	Mean	sd	Median	25 and 75 percentiles	Mean	sd	Median	25 and 75 percentiles	± %	*P*‡	Spearman correlation	Pearson EA correlation
Energy (kJ)	9249	2008	9172	7961, 10 498	8523	2429	8233	6652, 10 016	−7·9	0·014	0·41**	
Energy (kcal)	2211	480	2196	1903, 2509	2037	580	1967	1592, 2393	−7·9	0·014	0·41**	
Protein (g)	91	18	92	80, 101	71	22	64	58, 83	−22·0	<0·001	0·06	0·23
Fat (g)	85	18	81	71, 98	83	35	72	55, 105	−2·4	0·208	0·34**	0·26*
SFA (g)	34	11	31·7	26·6, 43·6	30	15	27	19, 37	−11·8	0·003	0·36**	0·32**
MUFA (g)	31	10	29·0	25·0, 35·0	31	14	28	21, 40	0	0·564	0·38**	0·36**
PUFA (g)	13	5	12·7	9·5, 16·5	15	6	14	11, 16	+15·4	0·105	0·22	0·32**
Carbohydrate (g)	234	66	223	200, 264	218	66	201	170, 263	−6·8	0·021	0·57**	0·45**
Dietary fibre (g)	22	8	21	18, 26	24	9	23	18, 28	+9·0	0·082	0·50**	0·38**
Alcohol (g)	14	15	9	1, 21	12	11	8·7	4·3, 15·9	−14·3	0·930	0·66**	0·65**
Whole grain wheat and rye (g)†	34	28	32	14, 48	48	28	47	27, 62	+41·2	<0·001	0·38**	0·35**
Retinol equivalents	928	552	791	590, 1048	891	496	780	563, 1100	−4·0	0·691	0·09	0·10
Vitamin C (mg)	110	139	87	53, 126	124	76	107	76, 145	+12·7	0·001	0·46**	0·22
Vitamin D (μg)	7·1	3·6	5·9	4·3, 9·7	7·9	4·1	6·8	5·4, 9·8	+11·3	0·297	0·05	0·24*
Vitamin E (mg)	10·6	3·4	10·2	8·4, 12·7	13·1	5·5	11·3	9·4, 14·9	+23·6	<0·001	0·35**	0·24*
Thiamin (mg)	1·51	0·51	1·5	1·2, 1·7	1·40	0·47	1·33	1·09, 1·63	−7·3	0·262	0·10	0·36**
Riboflavin (mg)	1·75	0·61	1·6	1·3, 2·0	1·66	0·65	1·48	1·18, 2·09	−5·1	0·365	0·31*	0·53**
Niacin equivalents (mg)	38·8	9·2	38·4	31·2, 43·8	31·7	9·5	29·5	25·2, 38·3	−18·3	<0·001	0·12	0·27*
Vitamin B_6_ (mg)	2·10	0·63	2·0	1·7, 2·4	1·91	0·62	1·76	1·47, 2·22	−9·0	0·037	0·21	0·36**
Vitamin B_12_ (μg)	6·83	4·13	5·9	4·7, 8·4	4·96	2·69	4·17	2·99, 6·14	−27·4	<0·001	0·06	0·20
Folate (μg)	279	126	242	216, 317	276	96	257	203, 324	−1·1	0·613	0·41**	0·47**
Fe (mg)	12·4	5·8	11·4	9·5, 14·2	13·1	4·9	12·1	10·1, 15·4	+5·6	0·079	0·44**	0·44**
Ca (mg)	935	300	908	751, 1125	774	363	691	472, 1107	−17·2	0·001	0·28*	0·23
Phyto-oestrogen intake, total (μg)	6094	5174	4515	2909, 6877	1527	726	1479	970, 1970	−74·9	<0·001	−0·02	−0·11
Lignan (μg)	6081	5173	4507	2905, 6871	1511	724	1474	961, 1944	−75·2	<0·001	−0·03	−0·11
Isoflavonoids (μg)	13	8	8	4, 15	16	16	10	6, 19	+23·1	0·045	0·42**	0·43**

* *P* *<* 0·05, ** *P* < 0·01.

† Whole grains derived only from wheat and rye.

‡ Wilcoxon signed-rank test.

We found a statistically significant correlation between the two methods for reported whole grain wheat and rye intake, as well as for intake of macronutrients, except for protein (Table 2). The lowest correlation coefficient was seen for lignans and the highest for alcohol intake. The correlation on isoflavonoid intake between the two methods was statistically significant (*r* 0·42; *P* *<* 0·001). The unadjusted nutrient intake correlation between the methods was on average *r* 0·29 (range −0·03 to 0·66) and the correlation coefficient was improved to *r* 0·30 (range −0·11 to 0·65) when energy-adjusted variables were used.

**Fig. 3.**
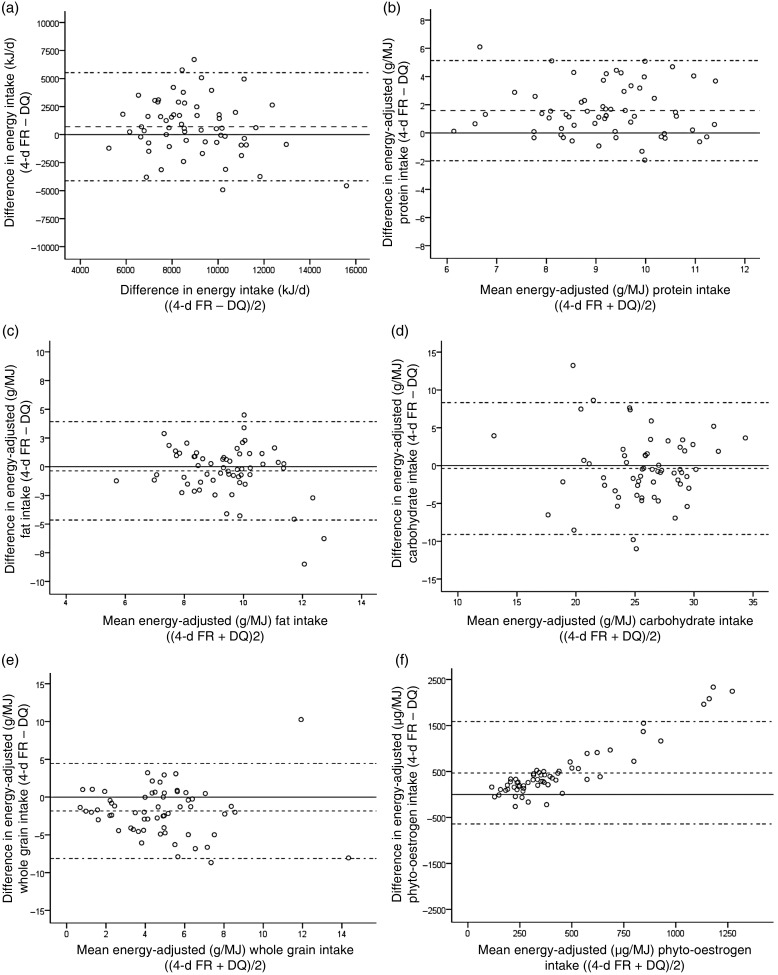
Bland–Altman plots comparing intakes measured with the 4-d food record (FR) and the dietary questionnaire (DQ): (a) energy; (b) protein; (c) fat; (d) carbohydrate; (e) whole grains; (f) phyto-oestrogens. The centre dashed line represents the mean difference; the top and bottom dashed lines represent ±1·96 sd.

Bland–Altman plots (Fig. 3) shows an acceptable agreement: most cases were within the 95 % limits of agreement on reported energy intake (Fig. 3(a)), as well as intake of carbohydrate (Fig. 3(d)), fat (Fig. 3(c)) and whole grains (Fig. 3(e)). Reported protein intake was underestimated in the DQ compared with the FR (Fig. 3(b)). Reported intake of phyto-oestrogens showed larger discrepancy at higher intakes, where intakes were predominantly higher and the intake distribution was skewed in the FR (Fig. 3(f)).

We found a statistically significant correlation between reported intake of whole grains (wheat and rye) measured with the DQ and levels of alkylresorcinol metabolites in urine (*r* 0·31; *P* = 0·015), but not with the FR (*r* 0·18; *P* = 0·12) (Table 3). Reported intake of phyto-oestrogens did not correlate with levels of alkylresorcinols. All analyses were also performed with Pearson partial correlations and adjusted for BMI, age, smoking status, fat and alcohol intake, but none of these variables affected the correlation coefficients significantly (data not shown).

**Table 3.** Cross-tabulation analysis for the proportion of individuals categorised in the same, adjacent or opposite tertile of dietary intake measured by 4-d food record (4-d FR) and diet questionnaire (DQ) or alkylresorcinol (AR) metabolites levels in urine

	Same (%)	Adjacent (%)	Opposite (%)	Weighted κ	Correlation
DQ					
Whole grain + AR total	44·4	47·6	8	0·29	0·31*
4-d FR					
Whole grain + AR total	36	49·2	14·8	0·11	0·18
DQ – 4-d FR					
Energy + energy	47·5	42·6	9·9	0·29	
Whole grain + whole grain	55·7	32·8	11·5	0·37	
Isoflavonoids + isoflavonoids	39·4	45·8	14·8	0·14	
Lignans + lignans	29·4	49·3	21·3	−0·04	
Phyto-oestrogens + phyto-oestrogens	29·4	49·3	21·3	−0·04	

* *P* *<* 0·05.

Results of cross-tabulation between the methods shows that the proportion of individuals categorised in the same tertile ranged from 29·4 % for lignans, to 55·7 % for whole grains, and the weighted κ values were −0·04 and 0·37, respectively (Table 3). Agreement on isoflavonoid intakes placed 39·4 % in the same tertile, with a weighted κ value of 0·14. The ranking of whole grains (wheat and rye) against alkylresorcinols performed better in the DQ than FR, with a weighted κ of 0·29 and 0·11, respectively.

## Discussion

In this study, the validity of a new web-based DQ was examined. Alkylresorcinol metabolites measured in urine were used as an objective biomarker of whole grain wheat and rye intake, and the results showed that the DQ had a satisfactory validity on whole grain wheat and rye assessment. Also, the DQ was in concordance with most nutrients compared with the FR. The questionnaire was perceived as easy to use and to understand according to the face-to-face validation.

### Strengths and limitations

A limitation of the study is that no objective biomarkers of phyto-oestrogen intake were used. However, to our knowledge, there are no adequate reference methods or biomarkers for measuring intake of phyto-oestrogens. We have previously investigated the correlation between lignan intake assessed with an FFQ and serum enterolactone levels, and found no correlation^(^^13^^)^. Low correlations between lignan intake and serum enterolactone levels have also been shown elsewhere^(^^20^^–^^23^^)^. These poor correlations are often attributed to the large individual variations in absorption, metabolism and differences in the gut microflora^(^^24^^–^^26^^)^. We therefore chose to validate the whole grain (wheat and rye) component in the questionnaire, of which there is a valid biomarker^(^^27^^,^^28^^)^. Although the sample size was relatively small (sixty-one individuals), a sample size of at least fifty individuals would be considered as sufficient when using biomarkers in dietary validation studies^(^^29^^)^.

In our study, the reference method of choice was a paper-based 4-d FR, because FFQ and FR have traditionally been considered to have unrelated measurement biases^(^^30^^)^. Compared with a paper-based assessment method, the administration of a web-based questionnaire is easier and reduces costs in terms of data collection and management^(^^31^^)^ (although errors in self-reported dietary intake measurement will occur in both instruments).

The cohort comprised men from the Swedish population register, as well as men having been diagnosed with prostate cancer, which enhances generalisation to the study population where the questionnaire is aimed to be used later. After conducting the face-to-face validation on the first ten study subjects, some small adjustments were made in the construction of the DQ to ensure that all questions were properly understood.

### Energy and nutrient assessment

Although the mean energy intake in the DQ was lower than in the FR, it is still in line with what other dietary surveys have reported in similar populations using paper-based FR^(^^32^^,^^33^^)^ and slightly higher than a study using an FFQ^(^^34^^)^. Reported energy intake was also consistent with the Swedish National Food Agency's nutrition survey ‘Riksmaten’ in 2010 for the same age group^(^^35^^)^. The lower energy intake was most likely to be derived from the low protein intake assessed in the DQ, and was demonstrated both by the low correlation coefficients against the reference method and by the Bland–Altman plots. It could be questioned if the portion sizes in the DQ on protein sources were too small, as standard portions would also be adapted for women. There might also be a lack of crucial protein sources in the food list. Our intention is to use the DQ in future studies and therefore we will identify and add important sources of protein to the food items list that we expect to be consumed in this population. Carbohydrate and fat intakes had statistically significant correlations between the methods and improved when energy-adjusted variables were used.

The largest discrepancy in reported intake was seen for lignans, which consequently also had the lowest correlation coefficient. By comparing intake of lignan-rich foods, which in both methods mainly consisted of rye bread and flaxseeds in this cohort, it is clear that the distribution for intake of these foods was highly skewed in the FR. This was demonstrated both by the large standard deviation in mean intake but also with the Bland–Altman plots with larger discrepancy at higher intake values, where intake was predominantly higher in the FR. The median intake of rye breads reported in the FR was also very high, 70 g/d in comparison with 43 g/d as displayed in the Riksmaten survey for men^(^^35^^)^. It could be that 4 d of recording is not long enough to be able to capture the habitual intake of rye bran and flaxseeds, and therefore a FR might not be a relevant measure for comparison of lignan intake. In a multicentre study, the European Prospective Investigation into Cancer and Nutrition (EPIC), phyto-oestrogen intake was estimated by a 24-h recall method. Among all study centres, the average intake was estimated to 2664 µg/d, and in the Swedish cohort to 1737–2089 µg/d^(^^36^^)^. These values are well in line with the phyto-oestrogen intake assessed with the DQ. Another FFQ, aiming to measure lignan intake, estimated the average intake to 1616 µg/d among Swedish women^(^^23^^)^. This is also well in line with the results from our DQ.

For isoflavonoids, the concordance between the DQ and the FR was better than for lignans. In the typical Swedish diet, major sources of lignan precursors are spread over more food items than sources of isoflavonoids, and are therefore more difficult to capture. For some food items, e.g. flaxseed and rye bran, not everyone may be aware of eating them because a wide variety of breads on the Swedish market contains some amount of rye brans and/or flaxseeds even though it might not be apparent. In contrast, it is more likely that a person is aware of having consumed a large amount of beans or soya products, which are rich sources of isoflavonoids, because they are not commonly used in Swedish products. Because of the large day-to-day variations in intake of foods rich in phyto-oestrogens, we believe that our DQ is more reliable for measuring phyto-oestrogen intake than a 4-d FR is. However, the DQ appears to be a better tool to measure intake of isoflavonoids than intake of lignans.

Bland–Altman plots on reported energy intake, as well as intake of carbohydrate, fat and whole grains, showed a good agreement between the methods, where most cases were within the limits of agreement.

### Assessment of whole grain wheat and rye intake – objective validation

Alkylresorcinol metabolites 3,5-dihydroxybenzoic acid (DHBA) and 3-(3,5-dihydroxyphenyl)-1-propanoic acid (DHPPA) were measured in urine and analysed according to standard methods^(^^16^^)^. To minimise the burden on study participants only spot urine was collected, that is, one urine sample collected in the morning rather than a 24-h urine collection. Urinary creatinine was therefore used as an adjustment for the concentration of urine in the biomarker analysis. This, however, introduces a potential source of error since creatinine levels can vary among individuals on a day-to-day basis^(^^37^^)^. Both the individual variation of creatinine secretion but also of alkylresorcinol metabolism can affect the outcome of the correlations. Alkylresorcinol metabolites measured in urine has the ability to reflect a short-term intake of whole grains^(^^38^^)^, and for that reason we expected urinary metabolites to correlate better with the FR because the urine sampling was conducted immediately after finishing the diet record. Our results showed the opposite, with a moderate yet statistical significant correlation between the reported intake of whole grains in the DQ against alkylresorcinols and a non-significant correlation against FR. When using concentration biomarkers as objective comparisons, a fully linear relationship is not to be expected^(^^39^^)^ (as in the case of recovery biomarkers, e.g. the doubly labelled water method for energy intake). No previous validation study has used urinary alkylresorcinol metabolites before, but they have shown to be as valid as plasma alkylresorcinol homologues with the advantage that collecting fasting urine samples are less invasive than taking blood samples^(^^40^^,^^41^^)^. A previous, although small, study validating an FFQ specifically designed to measure whole grain cereal food intake in the diet, using plasma alkylresorcinols as the biomarker, displayed a correlation of 0·54 on total whole grain intake and plasma alkylresorcinols^(^^42^^)^. Former studies have found correlations between 0·25 to 0·34 on rye bread and plasma alkylresorcinols^(^^43^^,^^44^^)^, and other intervention studies between 0·35 and 0·58 for whole grain intake and plasma alkylresorcinols^(^^45^^,^^46^^)^.

In nutrition epidemiology, dietary exposures are rarely assessed as absolute amounts but rather as the ranking of exposure, often in quartiles. Therefore, the ability to rank individuals on low, medium and high intake of whole grains was examined. According to the agreement with alkylresorcinols, a weighted κ coefficient of 0·29 was obtained which concludes that the ranking capacity can be considered as moderately good with the DQ^(^^47^^)^.

In conclusion, the results showed that the new DQ has a satisfactory validity for measuring intake of whole grains (wheat and rye) and most nutrients. Sources and portion sizes regarding protein intake need to be revised, but after these adjustments have been made, the DQ will be a promising tool.

## References

[1] Ferlay J, Steliarova-Foucher E, Lortet-Tieulent J, (2015) Reprint of: cancer incidence and mortality patterns in Europe: estimates for 40 countries in 2012. Eur J Cancer 51, 1201–1202. 10.1016/j.ejca.2012.12.027 23485231

[2] Westerlund A, Steineck G, Balter K, (2011) Dietary supplement use patterns in men with prostate cancer: the Cancer Prostate Sweden study. Ann Oncol 22, 967–972. 20926547 10.1093/annonc/mdq456

[3] Adlercreutz H, Mazur W, Bartels P, (2000) Phytoestrogens and prostate disease. J Nutr 130, 658S–659S. 10702603 10.1093/jn/130.3.658S

[4] Yan L & Spitznagel EL (2005) Meta-analysis of soy food and risk of prostate cancer in men. Int J Cancer 117, 667–669. 15945102 10.1002/ijc.21266

[5] Strom SS, Yamamura Y, Duphorne CM, (1999) Phytoestrogen intake and prostate cancer: a case–control study using a new database. Nutr Cancer 33, 20–25. 10227039 10.1080/01635589909514743

[6] Ewings P & Bowie C (1996) A case–control study of cancer of the prostate in Somerset and east Devon. Br J Cancer 74, 661–666. 8761387 10.1038/bjc.1996.418 PMC2074670

[7] Key TJ, Silcocks PB, Davey GK, (1997) A case–control study of diet and prostate cancer. Br J Cancer 76, 678–687. 9303371 10.1038/bjc.1997.445 PMC2228001

[8] Hebert JR, Hurley TG, Olendzki BC, (1998) Nutritional and socioeconomic factors in relation to prostate cancer mortality: a cross-national study. J Natl Cancer Inst 90, 1637–1647. 9811313 10.1093/jnci/90.21.1637

[9] Hedelin M, Balter KA, Chang ET, (2006) Dietary intake of phytoestrogens, estrogen receptor-β polymorphisms and the risk of prostate cancer. Prostate 66, 1512–1520. 16921512 10.1002/pros.20487

[10] Bonkhoff H & Berges R (2009) The evolving role of oestrogens and their receptors in the development and progression of prostate cancer. Eur Urol 55, 533–542. 19013008 10.1016/j.eururo.2008.10.035

[11] Hartman J, Strom A & Gustafsson JA (2012) Current concepts and significance of estrogen receptor β in prostate cancer. Steroids 77, 1262–1266. 22824289 10.1016/j.steroids.2012.07.002

[12] Lissner L, Lindroos AK & Sjostrom L (1998) Swedish Obese Subjects (SOS): an obesity intervention study with a nutritional perspective. Eur J Clin Nutr 52, 316–322. 9630380 10.1038/sj.ejcn.1600567

[13] Hedelin M, Klint A, Chang ET, (2006) Dietary phytoestrogen, serum enterolactone and risk of prostate cancer: the Cancer Prostate Sweden Study (Sweden). Cancer Causes Control 17, 169–180. 16425095 10.1007/s10552-005-0342-2

[14] Hedelin M, Lof M, Andersson TM, (2011) Dietary phytoestrogens and the risk of ovarian cancer in the women's Lifestyle and Health Cohort Study. Cancer Epidemiol Biomarkers Prev 20, 308–317. 21098648 10.1158/1055-9965.EPI-10-0752

[15] Kost och Näringsdata AB (2015) Diet and Nutrition Data. Computer programs for nutrition calculation. http://www.kostdata.se/en (accessed August 2016).

[16] Marklund M, Landberg R, Aman P, (2010) Determination of alkylresorcinol metabolites in human urine by gas chromatography-mass spectrometry. J Chromatogr B Analyt Technol Biomed Life Sci 878, 888–894. 10.1016/j.jchromb.2010.02.007 20223713

[17] Willet W (1998) Nutritional Epidemiology, 2nd ed. New York: Oxford University Press.

[18] Bland JM & Altman DG (1986) Statistical methods for assessing agreement between two methods of clinical measurement. Lancet i, 307–310. 2868172

[19] Statistics Sweden (2013) Fördelning: Födelseland, kön och åldersgrupp i kombination med utbildningsnivå. http://www.scb.se/en_/Finding-statistics/ (accessed July 2013).

[20] Bhakta D, Higgins CD, Sevak L, (2006) Phyto-oestrogen intake and plasma concentrations in South Asian and native British women resident in England. Br J Nutr 95, 1150–1158. 16768838 10.1079/bjn20061777

[21] Durazzo A, Carcea M, Adlercreutz H, (2014) Effects of consumption of whole grain foods rich in lignans in healthy postmenopausal women with moderate serum cholesterol: a pilot study. Int J Food Sci Nutr 65, 637–645. 24611636 10.3109/09637486.2014.893283

[22] Milder IE, Kuijsten A, Arts IC, (2007) Relation between plasma enterodiol and enterolactone and dietary intake of lignans in a Dutch endoscopy-based population. J Nutr 137, 1266–1271. 17449591 10.1093/jn/137.5.1266

[23] Lin Y, Wolk A, Hakansson N, (2013) Validation of FFQ-based assessment of dietary lignans compared with serum enterolactone in Swedish women. Br J Nutr 109, 1873–1880. 23006454 10.1017/S000711451200387X

[24] Kilkkinen A, Stumpf K, Pietinen P, (2001) Determinants of serum enterolactone concentration. Am J Clin Nutr 73, 1094–1100. 11382665 10.1093/ajcn/73.6.1094

[25] Yoder S, Lancaster S, Hullar MA, (2014) Gut microbial metabolism of plant lignans: influence on human health In Diet–Microbe Interactions in the Gut: Effects on Human Health and Disease, pp. 103–117 [K Tuohy and D Del Rio, editors]. London: Academic Press.

[26] Lampe JW (2003) Isoflavonoid and lignan phytoestrogens as dietary biomarkers. J Nutr 133, Suppl. 3, 956s–964s. 12612182 10.1093/jn/133.3.956S

[27] Landberg R, Aman P, Friberg LE, (2009) Dose response of whole-grain biomarkers: alkylresorcinols in human plasma and their metabolites in urine in relation to intake. Am J Clin Nutr 89, 290–296. 19056600 10.3945/ajcn.2008.26709

[28] McKeown NM, Marklund M, Ma J, (2016) Comparison of plasma alkylresorcinols (AR) and urinary AR metabolites as biomarkers of compliance in a short-term, whole-grain intervention study. Eur J Nutr 55, 1235–1244. 26043861 10.1007/s00394-015-0936-8

[29] Serra-Majem L, Andersen LF, Henríque-Sánchez P, (2009) Evaluating the quality of dietary intake validation studies. Br J Nutr 102, S3–S9. 20100366 10.1017/S0007114509993114

[30] Cade J, Thompson R, Burley V, (2002) Development, validation and utilisation of food-frequency questionnaires – a review. Public Health Nutr 5, 567–587. 12186666 10.1079/PHN2001318

[31] Illner AK, Freisling H, Boeing H, (2012) Review and evaluation of innovative technologies for measuring diet in nutritional epidemiology. Int J Epidemiol 41, 1187–1203. 22933652 10.1093/ije/dys105

[32] Ax E, Garmo H, Grundmark B, (2014) Dietary patterns and prostate cancer risk: report from the population based ULSAM cohort study of Swedish men. Nutr Cancer 66, 77–87. 24325263 10.1080/01635581.2014.851712

[33] Goluch-Koniuszy Z, Rygielska M & Nowacka I (2013) Nutritional status and nutritional habits of men with benign prostatic hyperplasia or prostate cancer – preliminary investigation. Acta Sci Pol Technol Aliment 12, 319–330. 24584961

[34] Vidal AC, Williams CD, Allott EH, (2015) Carbohydrate intake, glycemic index and prostate cancer risk. Prostate 75, 430–439. 25417840 10.1002/pros.22929 PMC4293225

[35] Livsmedelsverket (2013) Riksmaten – vuxna 2010–2011. Livsmedels- och näringsintag bland vuxna I Sverige: Livsmedelsverket, Uppsala 2012. http://www.slv.se/sv/grupp1/Mat-och-naring/Matvanor---undersokningar/ (accessed April 2013).

[36] Zamora-Ros R, Knaze V, Lujan-Barroso L, (2012) Dietary intakes and food sources of phytoestrogens in the European Prospective Investigation into Cancer and Nutrition (EPIC) 24-hour dietary recall cohort. Eur J Clin Nutr 66, 932–941. 22510793 10.1038/ejcn.2012.36

[37] Alessio L, Berlin A, Dell'Orto A, (1985) Reliability of urinary creatinine as a parameter used to adjust values of urinary biological indicators. Int Arch Occup Environ Health 55, 99–106. 3988361 10.1007/BF00378371

[38] Soderholm PP, Koskela AH, Lundin JE, (2009) Plasma pharmacokinetics of alkylresorcinol metabolites: new candidate biomarkers for whole-grain rye and wheat intake. Am J Clin Nutr 90, 1167–1171. 19759167 10.3945/ajcn.2009.28290

[39] Jenab M, Slimani N, Bictash M, (2009) Biomarkers in nutritional epidemiology: applications, needs and new horizons. Hum Genet 125, 507–525. 19357868 10.1007/s00439-009-0662-5

[40] Marklund M, Landberg R, Andersson A, (2013) Alkylresorcinol metabolites in urine correlate with the intake of whole grains and cereal fibre in free-living Swedish adults. Br J Nutr 109, 129–136. 22470195 10.1017/S0007114512000621

[41] Aubertin-Leheudre M, Koskela A, Samaletdin A, (2010) Plasma and urinary alkylresorcinol metabolites as potential biomarkers of breast cancer risk in Finnish women: a pilot study. Nutr Cancer 62, 759–764. 20661824 10.1080/01635581003693058

[42] Ross AB, Pineau N, Kochhar S, (2009) Validation of a FFQ for estimating whole-grain cereal food intake. Br J Nutr 102, 1547–1551. 19622189 10.1017/S0007114509990845

[43] Linko AM, Juntunen KS, Mykkanen HM, (2005) Whole-grain rye bread consumption by women correlates with plasma alkylresorcinols and increases their concentration compared with low-fiber wheat bread. J Nutr 135, 580–583. 15735097 10.1093/jn/135.3.580

[44] Landberg R, Kamal-Eldin A, Aman P, (2011) Determinants of plasma alkylresorcinol concentration in Danish post-menopausal women. Eur J Clin Nutr 65, 94–101. 20859297 10.1038/ejcn.2010.193

[45] Landberg R, Kamal-Eldin A, Andersson A, (2008) Alkylresorcinols as biomarkers of whole-grain wheat and rye intake: plasma concentration and intake estimated from dietary records. Am J Clin Nutr 87, 832–838. 18400704 10.1093/ajcn/87.4.832

[46] Ross AB, Bourgeois A, Macharia HN, (2012) Plasma alkylresorcinols as a biomarker of whole-grain food consumption in a large population: results from the WHOLEheart Intervention Study. Am J Clin Nutr 95, 204–211. 22170369 10.3945/ajcn.110.008508 PMC3592483

[47] Landis JR & Koch GG (1977) The measurement of observer agreement for categorical data. Biometrics 33, 159–174. 843571

